# Antiapoptotic and Antiautophagic Effects of Eicosapentaenoic Acid in Cardiac Myoblasts Exposed to Palmitic Acid

**DOI:** 10.3390/nu4020078

**Published:** 2012-02-07

**Authors:** Silvia Cetrullo, Benedetta Tantini, Flavio Flamigni, Claudia Pazzini, Annalisa Facchini, Claudio Stefanelli, Claudio M. Caldarera, Carla Pignatti

**Affiliations:** 1 Department of Biochemistry “G. Moruzzi”, University of Bologna, via Irnerio 48, Bologna 40126, Italy; Email: benedetta.tantini@unibo.it (B.T.); flavio.flamigni@unibo.it (F.F.); claudia.pazzini@studio.unibo.it (C.P.); annalisa.facchini@unibo.it (A.F.); claudio.stefanelli@unibo.it (C.S.); carla.pignatti@unibo.it (C.P.); 2 National Institute for Cardiovascular Research, via Irnerio 48, Bologna 40126, Italy; Email: claudio.caldarera@unibo.it

**Keywords:** eicosapentaenoic acid, palmitic acid, apoptosis, autophagy, H9c2 cardiomyoblasts

## Abstract

Apoptosis is a programmed cell death that plays a critical role in cell homeostasis. In particular, apoptosis in cardiomyocytes is involved in several cardiovascular diseases including heart failure. Recently autophagy has emerged as an important modulator of programmed cell death pathway. Recent evidence indicates that saturated fatty acids induce cell death through apoptosis and this effect is specific for palmitate. On the other hand, *n*-3 polyunsaturated fatty acids (PUFAs) have been implicated in the protection against cardiovascular diseases, cardiac ischemic damage and myocardial dysfunction. In the present study we show that *n*-3 PUFA eicosapentaenoic acid (EPA) treatment to culture medium of H9c2 rat cardiomyoblasts protects cells against palmitate-induced apoptosis, as well as counteracts palmitate-mediated increase of autophagy. Further investigation is required to establish whether the antiautophagic effect of EPA may be involved in its cytoprotective outcome and to explore the underlying biochemical mechanisms through which palmitate and EPA control the fate of cardiac cells.

## 1. Introduction

It is universally recognized that the quality and quantity of dietary fat ingested may contribute to the onset and progression of several chronic diseases impacting human health. Excessive levels of dietary saturated fatty acids or an imbalance of saturated *versus* unsaturated fats have been implicated in the pathogenesis of diabetes, obesity, atherosclerosis and cardiovascular diseases [1]. High fat feeding increases fat mass, elevates plasma free fatty acid levels and suppresses insulin signaling [2]. Elevated plasma levels of fatty acids represent a risk factor for coronary artery disease and cardiac dysfunction. Moreover, accumulation of saturated fatty acids in the heart has been involved in the development of heart failure and cardiomyopathy [3,4].

On the other hand, long chain *n*-3 polyunsaturated fatty acids (PUFAs), in which the first double bond is located between carbon 3 and 4, counting from methyl end (*omega* or *n*) of the carbon chain, proved to exert beneficial effects on the cardiovascular system. Overall research indicated that the consumption of seafood or marine-derived (eicosapentaenoic acid, EPA; docosahexaenoic acid, DHA) *n*-3 PUFAs reduces coronary heart disease mortality in populations with or without established cardiovascular diseases [5]. Recent studies have shown that *n*-3 PUFA can prevent myocardial tissue derangement in hypertrophic cardiomyopathic hamsters improving heart function and dramatically extending the cardiomyopathic animal longevity [6]. Nonetheless, the mechanisms through which *n*-3 PUFA beneficial effects on the cardiac tissues occur remain largely unknown. In response to pathophysiological stresses, cardiac myocytes undergo hypertrophic growth and/or apoptosis, which are associated to the development of cardiac pathologies. Accumulating evidence from *in vivo* and *in vitro* studies strongly suggests that apoptosis may play an important role in the pathogenesis of several cardiovascular diseases, such as ischemia/reperfusion, infarction, heart failure and aging [7].

It has recently become clear that different naturally occurring dietary fatty acids are able to modulate the molecular pathways involved in apoptosis. Palmitic acid has been implicated in the induction of apoptosis in different cell types, including neonatal cardiomyocytes. In these cells EPA supplementation is able to modulate gene expression [8] and elicits a protective effect against apoptosis induced by saturated fatty acid [9].

Recent studies have shown that autophagy modulates whether cardiomyocytes go towards survival or cell death. Autophagy is a catabolic process whereby cells respond to energy stress by degrading and recycling damaged proteins and intracellular components: ribosomes, lipids and even entire organelles [10,11]. Physiologically, autophagy functions as a quality control of cellular milieu, however, dysregulation of autophagy is implicated in a wide variety of pathological conditions and can lead to cell death [12,13]. Autophagy plays a role in the pathogenesis of a number of human diseases, and conditions including obesity and steatosis [14]. It is however unknown what factor or factors determine whether autophagy will be protective or detrimental to the cell, it is likely that the level and duration of autophagy are important.

A complex interrelationship seems to exist between autophagy and the apoptotic cell death. The cross-talk between the two processes is sometimes contradictory, but surely critical to the overall fate of the cell (reviewed in ref. [15]). Recently autophagy has been proposed as a novel mechanism involved in the protective action of resveratrol, a nutritional factor [16]. However to our knowledge, no evidence is available regarding the influence of other cardioprotective nutrients, such as PUFAs, on autophagy. 

In the present study we have evaluated the effect of palmitic acid and EPA on the induction of cell death and autophagy in cardiac cells. We have found that palmitic acid (at concentrations normally found in plasma during fasting conditions) induces both autophagy and cell death by apoptosis, whereas EPA exerts a clear protective effect against palmitate toxicity.

## 2. Experimental Section

### 2.1. Materials

Monoclonal anti-microtubule-associated protein 1A/1B-light chain 3 (LC3) and anti-glyceraldehyde 3-phosphate dehydrogenase (GAPDH) and secondary anti-rabbit antibodies were purchased from Cell Signaling Technology. Enhanced chemiluminescence (ECL) detection system was provided by GE Healthcare. DNA breaks detection was performed using the DeadEnd™ Assays obtained from Promega. All other chemicals were from Sigma Aldrich.

### 2.2. Cell Cultures

H9c2 embryonal rat-heart derived cells were cultured in Dulbecco’s Modified Eagle Medium supplemented with 10% heat inactivated fetal bovine serum, 100 IU/mL penicillin and 0.1 µg/mL streptomycin. For experiments, subcultured cells were grown for 48 h, than fresh medium was supplied with or without fatty acid addition. 

### 2.3. Fatty Acid Supplementation

Palmitate 5 mM stock solution was prepared with 10% fatty acid-free albumin from bovine serum (BSA) and 5% ethanol. 10 mM EPA stock solution in absolute ethanol was mixed with 150 µM BSA, and the final concentrations in the medium were 60 µM EPA and 30 µM BSA. Control cells were treated with the same BSA and ethanol solutions without palmitate or EPA. 

### 2.4. Cell Viability

Evaluation of viable cells in our experimental protocol was performed by two different methods: the 3-(4,5-dimethylthiazol-2-yl)-2,5-diphenyltetrazolium bromide (MTT) assay and Trypan Blue exclusion test. The first is a colorimetric method based on the ability of viable cells to reduce MTT from a yellow water-soluble dye to a dark blue insoluble formazan product [17]. The Trypan Blue exclusion method is based on the count with a Burker hemocytometer of both living cells and stained dead cells. Dead cells were reported as a percentage of the total number of cells. 

### 2.5. Determination of Caspase Activity

Caspase activity was measured by the cleavage of the fluorogenic peptide Ac-Asp-Glu-Val-Asp-7-amido-4-methylcoumarin (Ac-DEVD-AMC), which represents a substrate for caspase-3 and other effector caspases, as previously described [18,19]. 

### 2.6. DNA Breaks Detection by *in Situ*-Labeling and Nuclear Staining

To label apoptotic nuclei, the 3′-OH end of DNA fragments was visualized by the method of terminal transferase-mediated dUTP nick end-labeling (TUNEL). The nuclei of apoptotic and non-apoptotic cells were counterstained with 0.1 μg/mL 4′,6-diamidino-2-phenylindole (DAPI). The labeled cells were analyzed by fluorescence microscopy. The percentage of apoptotic cells was calculated as the ratio of the number of TUNEL-positive cells to the total number of DAPI-stained cells, counted in three different random fields.

### 2.7. Immunoblot Analysis

The proteins were detected in cell extracts by Western blotting: equal amounts of cell extract were subjected to electrophoresis in 15% gels, blotted onto nitrocellulose membranes as described [20], and probed with anti-LC3 antibody or anti-GAPDH antibody at 4 °C overnight. After washes, membranes were incubated with horseradish peroxidase-conjugated anti-rabbit IgG for 1 h. The chemiluminescent signals were detected using an ECL system. GAPDH was used as loading control. A representative image of visualized immunoreactive bands and densitometric analysis show the relative intensity of LC3-II expression.

### 2.8. Statistical Analysis

All the data presented as graphs are expressed as means ± SEM of the indicated numbers of determinations, and analyzed for statistical significance by one-way or two-way ANOVA. Differences were considered significant for *P* < 0.05.

## 3. Results

### 3.1. Palmitate Induces Cell Death in H9c2: Effect of EPA

In order to determine the effects of saturated fatty acids on myocardial cells, we first tested the viability of H9c2 cardiomyoblasts treated with increasing doses of palmitate. 

**Figure 1:**
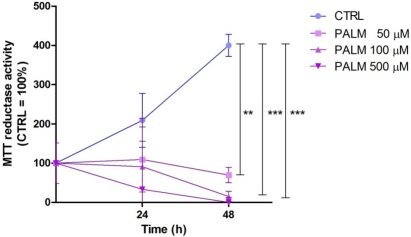
Effect of palmitate on H9c2 cardiomyoblasts viability. H9c2 cells were incubated for the indicated times under control conditions (CTRL) or increasing concentrations of palmitate (PALM). Results are means ± SEM. **P < 0.005; ***P < 0.001.

Figure 1 shows that under control conditions, palmitate (50–500 µM) significantly and time-dependently decreased cell viability, assessed by the MTT reduction assay. Since palmitate was most effective in reducing the cell viability at the dose of 500 µM, we used this concentration in all subsequent experiments.

**Figure 2:**
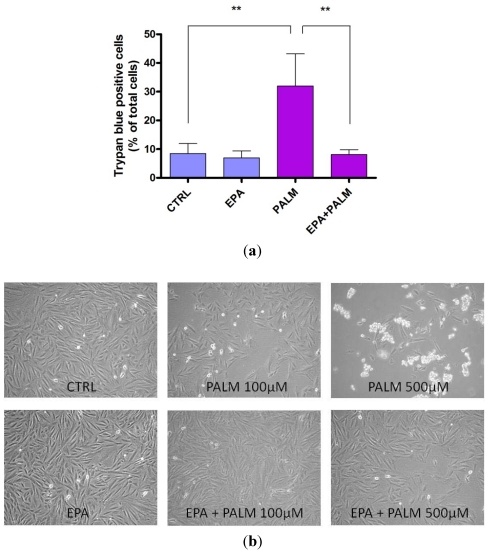
Effect of eicosapentaenoic acid (EPA) on survival of H9c2 exposed to palmitate. (**a**) The survival of cells exposed to 500 µM palmitate in the presence or absence of 60 μM EPA was measured by Trypan Blue exclusion. Results are means ± SEM of four determinations. **P < 0.005; (**b**) Representative pictures of dying H9c2 cells exposed to different doses of palmitate. H9c2 cells were incubated for 24 h under control condition (CTRL), in the presence of different concentrations of palmitate (as shown in the picture), or palmitate plus 60 μM EPA.

The palmitate-mediated reduction of cell viability was also confirmed by Trypan Blue exclusion test (Figure 2a). In particular, after a 24 h treatment with palmitate, the number of cells that included Trypan Blue increased to about 30% of total cells compared to 10% of control.

Long chain PUFAs play an important role in cardioprotection. These effects have been largely attributed to membrane DHA. Unlike DHA, EPA does not significantly enter cardiac membranes *in vivo* [21] and its beneficial effects have been often attributed to an acute effect [22]. 

In our experimental conditions a protective effect of EPA on palmitate reduction of H9c2 viability was observable when the polyunsaturated fatty acid was added to the cells together with palmitate. In fact, Figure 2a shows that while the treatment of H9c2 cardiomyoblasts with 60 µM EPA did not affect cell survival in control conditions, it significantly reduced cell death in palmitate-treated cultures. 

The protective effect of EPA is also depicted in the pictures in Figure 2b which shows that EPA attenuated the palmitate increase in cell death. The pro-survival effect of EPA was particularly evident when 500 µM palmitate was used to induce cell death. 

### 3.2. Protective Effect of EPA on Palmitate-Induced Apoptosis

In order to determine whether the observed reduction in viability of palmitate-treated cardiomyoblasts was due to apoptosis, H9c2 cells were treated with palmitate and caspase activity or TUNEL assay were used as markers of apoptosis.

**Figure 3:**
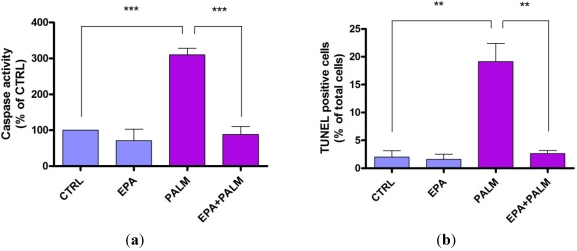
Effect of EPA on apoptosis of H9c2 cardiomyoblasts exposed to palmitate. 48 h after plating H9c2 cells were incubated for 24 h with or without 500 µM palmitate in the presence or absence of 60 µM EPA. Cells were then collected for subsequent analysis. (**a**) The cells were assayed for caspase activity hydrolyzing the peptide sequence DEVD (DEVDase activity). The data are means ± SEM of four determinations. ***P < 0.001. (**b**) DNA fragmentation was determined by the quantitative assay of apoptotic cells (TUNEL assay). The data are means ± SEM of three determinations. **P < 0.005.

Figure 3a shows that after 24 h treatment with palmitate, caspase activity was increased about 3 times compared to control cultures. Figure 3a also shows that EPA treatment did not affect the basal caspase-3 activity, but abolished the caspase-3 activation due to palmitate. 

The antiapoptotic effect of EPA was also evident from its ability to counteract DNA fragmentation in H9c2 cells exposed to palmitate, as determined by TUNEL assay (Figure 3b). These results show that the treatment with EPA counteracts the main biochemical markers of apoptosis such as the activation of caspases and the DNA fragmentation triggered by palmitate, thus suggesting that EPA may exert a protective effect in H9c2 cardiomyoblasts by preventing apoptotic programs. 

### 3.3. Palmitate Treatment Induces Autophagy in H9c2 Cardiac Cells: Effect of EPA

The process of autophagosome formation depends on several autophagy proteins. SDS-PAGE and immunoblotting permit to detect endogenous LC3 as two bands: one represents LC3 I, which is the cytosolic form, and the other LC3 II, which is conjugated with phosphatidylethanolamine, and is membrane bound in autophagosomes [23].

**Figure 4:**
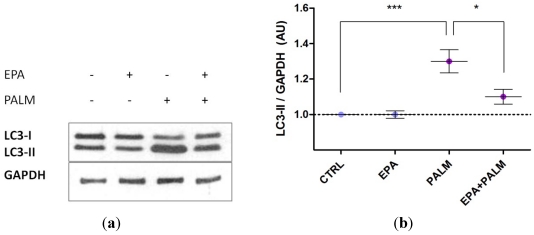
Effect of palmitate and EPA on H9c2 autophagy. (**a**) 48 h after plating H9c2 cells were incubated for 4 h with or without 500 µM palmitate in the presence or the absence of 60 µM EPA. Cell extracts were prepared and analyzed by Western blotting for cleavage of LC3 protein; GAPDH was used as internal control; (**b**) Densitometric analysis on immunoblots of six independent experiments was performed. ***P < 0.001; *P < 0.05.

In our experimental conditions we found that palmitate induced autophagy. In fact, as shown in Figure 4a, when H9c2 cardiomyoblasts were treated for 4 h with palmitate, a strong increase in the expression of LC3-II, an indicator of autophagosome formation, was observed (lane 3). This effect was completely prevented when EPA was added to the cells together with palmitate (lane 4). Quantification of the bands by densitometry is also shown (Figure 4b).

In order to determine whether autophagy plays a role in the induction of apoptosis under our experimental conditions, we treated cell cultures with the specific autophagy inhibitor 3-methyladenine (3-MA).

**Figure 5:**
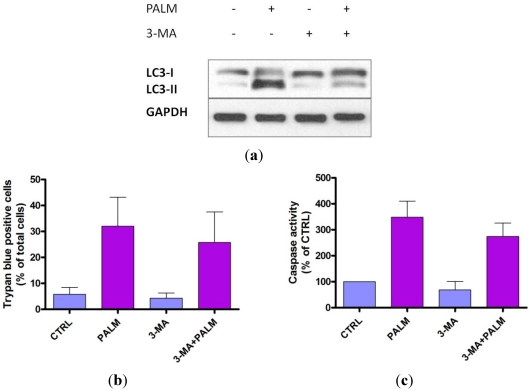
Effect of autophagy inhibition on cell death induced by palmitate. (**a**) 48 h after plating, H9c2 cells were incubated for 4 h with or without 500 µM palmitate in the presence or the absence of 10 µM 3-MA. Cell extracts were prepared and analyzed by Western blotting for cleavage of LC3 protein; GAPDH was used as internal control; (**b**,**c**) The cells exposed to 500 µM palmitate for 24 h in presence or absence of 10 μM 3-MA were assayed for caspase activity and Trypan Blue exclusion. Results are means ± SEM of three determinations.

Figure 5a shows that the treatment of H9c2 cultures with 3-MA significantly prevented palmitate-induced autophagy, evidenced by a decreased expression of protein LC3-II.

Figure 5b,c also show that palmitate-induced cell death, as indicated by Trypan Blue exclusion method or caspase activation, was not significantly modified in the presence of 3-MA, thus suggesting that autophagy might not be involved in the execution of apoptosis in these cells.

## 4. Discussion

Maintenance of cell viability is critically important, especially in myocytes, since their viability determines cardiac performance which, when impaired, leads to heart failure. Of the various inducers of cell death, saturated fatty acids have been recently reported to induce apoptosis in many different cell types [9,24], while EPA is believed to have a protective action against cardiovascular diseases, particularly coronary artery diseases [6].

Experimental evidence has shown that high levels of circulating saturated fatty acids are associated with coronary artery disease and cardiac dysfunction. In particular the accumulation of saturated fatty acids in the heart has been proposed to play a role in the development of heart failure and cardiomyopathy [3,4]. Clinical data show a correlation between fatty acid plasma level and cardiovascular disease, and several studies partially explained mechanisms by which saturated fatty acids directly induce lipotoxicity.

In the present paper we showed that H9c2 cells exposed to palmitate display a reduced viability and an increase in caspase activation and DNA fragmentation, the main biochemical markers of apoptosis. The palmitate-induced damage can be related with overproduction of ceramides that release cytotoxic free radicals and cytochrome *c* from the mitochondria, thus provoking apoptotic cell death [25,26]. Moreover palmitic acid can alter the levels of the cardiomyocyte cardiolipin, an important component of mitochondrial inner membrane [27]. It has also been reported that ischemic events promote fatty acid flux to the cardiac cells, increasing oxidative stress and contributing to cardiomyocyte damage [28]. 

Numerous epidemiologic and observational studies report beneficial effects of *n*-3 PUFAs in the primary prevention, coronary artery disease and post-myocardial infarction, sudden cardiac death, heart failure and atherosclerosis [5,29]. However, their role in cardiac arrhythmias is still controversial. Some publications, in fact, supported a pro-arrhythmic effect of *n*-3 fatty acids [30,31,32], whereas many studies suggest that the modulation of ion channels exerted by PUFAs in animal models [22] would lead them to have anti-arrhythmic effects.

Although PUFAs have an overall clinical protective role in cardiac patients, whether *n*-3 fatty acids exert direct effects on cardiac cell life has not been elucidated, in particular when it is challenged by noxious stimuli, such as following ingestion of diets too high in saturated fatty acid, during atherosclerosis, myocardial ischemia and heart failure.

Our results showed a protective effect of EPA against palmitate-induced apoptosis and are consistent with other studies performed in different cell types [33]. In particular it has been recently reported that EPA supplementation protected rat neonatal cardiomyocytes against palmitate-induced apoptosis via the implication of several mitochondrial elements such as the translocation of Bax to the mitochondria and the release of cytochrome *c* [9].

According to recent studies, autophagy has emerged as a physiological process implicated in a wide variety of pathological conditions. In the presence of ample nutrient supply, anabolic reactions predominate within the cell and autophagy is maintained at low levels, those critical for normal cellular homeostasis and survival [34]. At the present new evidence suggests that autophagy can have a dual role in the heart. This process is rapidly activated in cardiac myocytes in response to a starvation period [35]. Upregulation of autophagy also occurs in the presence of external stressors, such as hormonal imbalance, hypoxia, oxidative stress and mitochondrial dysfunction [36,37,38]. Moreover, enhanced levels of autophagy are observed in certain clinically important circumstances, including neurodegenerative disorders, microbial invasion and cardiovascular diseases [12,13,39]. In particular, cardiac hypertrophy and the transition from hypertrophy to heart failure are modulated by an autophagic machinery [36].

In our experimental model we found that palmitate induces autophagy. This result prompted us to suggest that in palmitate-treated H9c2 cells, both apoptosis and autophagy can act as partners to induce cell death in a coordinated and cooperative manner, as shown in other experimental models [15]. The treatment with EPA showed an evident protective effect against palmitate induced toxicity, but it also prevented autophagy induction as well as apoptotic cell death. On the other hand, in a preliminary experiment with the autophagy inhibitor 3-MA, a significant cause-effect relationship between the two events was not observed.

Although a direct and exclusive relationship between autophagy and apoptosis induction following treatment with palmitate might not exist, we cannot exclude that autophagy inhibition by EPA could be involved in potentiating the clear protective effect against palmitic acid-induced cell death and apoptosis in this cell model. 

## 5. Conclusions

In the present study we have evidenced that palmitic acid produces a cytotoxic effect on H9c2 cardiomyoblasts and induces cell death by apoptosis. Interestingly, these effects are preceded and paralleled by an evident appearance of autophagy. The presence of EPA in the culture medium is able to counteract the pro-apoptotic and pro-autophagic effect of palmitate, so exerting a protective action in H9c2 cells. A full understanding of the multifaceted relationship between apoptosis and autophagy will be critical for the assessment of novel approaches for the treatment of cardiomyocyte injury associated with myocardial apoptosis. Detailing the molecular mechanisms of the process is of importance in order to then identify therapeutic targets that can be used to address cardiac disease. In conclusion, these findings provide further insight as to the cardiotoxic effect of saturated fatty acids and give important clinical implications regarding the positive role of *n*-3 PUFAs in reducing the risk of cardiovascular diseases related to cardiac cell death.
